# eHealth Engagement as a Response to Negative Healthcare Experiences: Cross-Sectional Survey Analysis

**DOI:** 10.2196/11034

**Published:** 2018-12-05

**Authors:** Nicole Senft, Jordan Everson

**Affiliations:** 1 Department of Medicine Vanderbilt University Medical Center Nashville, TN United States; 2 Department of Health Policy Vanderbilt University School of Medicine Nashville, TN United States

**Keywords:** care coordination, eHealth, health disparities, patient-centered care

## Abstract

**Background:**

eHealth provides individuals with new means of accessing health information and communicating with providers through online channels. Prior evidence suggests that patients use eHealth to find information online when they receive care that is low in patient centeredness. However, it is unclear how other problems with the healthcare-delivery system motivate the use of eHealth, how these problems relate to different kinds of eHealth activities, and which populations are most likely to use eHealth when they receive low-quality care.

**Objective:**

We aimed to determine how two types of negative care experiences—low patient centeredness and care coordination problems—motivate the use of different eHealth activities, and whether more highly educated individuals, who may find these tools easier to use, are more likely to use eHealth following negative experiences than less highly educated individuals.

**Methods:**

Using nationally representative data from the 2017 Health Information National Trends Survey, we used factor analysis to group 25 different eHealth activities into categories based on the correlation between respondents’ reports of their usage. Subsequently, we used multivariate negative binomial generalized linear model regressions to determine whether negative healthcare experiences predicted greater use of these resulting categories. Finally, we stratified our sample based on education level to determine whether the associations between healthcare experiences and eHealth use differed across groups.

**Results:**

The study included 2612 individuals. Factor analysis classified the eHealth activities into two categories: provider-facing (eg, facilitating communication with providers) and independent (eg, patient-driven information seeking and communication with non-providers). Negative care experiences were not associated with provider-facing eHealth activity in the overall population (care coordination: *P*=.16; patient centeredness: *P*=.57) or among more highly educated respondents (care coordination: *P*=.73; patient centeredness: *P*=.32), but respondents with lower education levels who experienced problems with care coordination used provider-facing eHealth more often (IRR=1.40, *P*=.07). Individuals engaged in more independent eHealth activities if they experienced problems with either care coordination (IRR=1.15 *P*=.01) or patient-centered communication (IRR=1.16, *P*=.01). Although care coordination problems predicted independent eHealth activity across education levels (higher education: IRR=1.13 *P*=.01; lower education: IRR=1.19, *P*=.07), the relationship between low perceived patient centeredness and independent activity was limited to individuals with lower education levels (IRR=1.25, *P*=.02).

**Conclusions:**

Individuals use a greater number of eHealth activities, especially activities that are independent of healthcare providers, when they experience problems with their healthcare. People with lower levels of education seem particularly inclined to use eHealth when they have negative healthcare experiences. To maximize the potential for eHealth to meet the needs of all patients, especially those who are traditionally underserved by the healthcare system, additional work should be performed to ensure that eHealth resources are accessible and usable to all members of the population.

## Introduction

eHealth is defined as “health services and information delivered or enhanced through the Internet and related technologies” [1] and provides patients with a set of tools to engage in their health and healthcare. Along with the increase in provider use of electronic health records and associated tools over the past decade, the variety of eHealth tools available to patients has also increased [2-4]. eHealth resources may allow patients to more actively engage in their health and address problems unaddressed by their providers [5,6]. For example, patients can seek health information online that was not provided or was poorly provided by their care provider; in addition, they can use secure messaging to ensure their test results are seen by specific providers when they perceive poor coordination among their care team.

Existing evidence indicates that patients who experience deficits in the patient-provider relationship are more likely to seek health information online than those who do not experience such deficits [7-10]. For example, Li and colleagues [7] found that 40% of patients sought information online because they believed their doctor had provided them with inaccurate or incomplete information, or that the doctor’s care was not as good as it should have been. In particular, patients who rated their physician as having low patient-centered communication (ie, communication that is respectful of and responsive to individual patients’ needs and preferences [11]) were more likely to seek information online following their appointments, suggesting that online health information may help patients meet informational needs that are not adequately met within the patient-provider relationship. However, the focus in existing research on the association between patients’ perceptions of providers and health information seeking does not clarify how dissatisfaction with care might relate to other kinds of eHealth tools. In particular, tools that provide a means to communicate with healthcare providers (eg, secure messaging) may meet different needs from tools that provide access to information and support that is relatively independent of the healthcare system (eg, health information seeking). In addition, evidence on the impact of other negative healthcare experiences beyond low patient centeredness on eHealth use is limited. No studies have thus far examined the association between care coordination and the use of eHealth. Care coordination problems reflect a system-level failure to organize patient-care activities across multiple people or organizations. Patients may perceive this problem in a different way than they perceive problems in the patient-provider relationship and may use different kinds of eHealth resources in efforts to facilitate coordination of their care.

Patients’ use of the Internet for health-related reasons varies according to the individual’s needs [12,13]. It is therefore likely that different kinds of problematic healthcare experiences are associated with the use of different eHealth tools. Understanding which eHealth resources can be categorized together based on their use and how healthcare experiences predict different kinds of eHealth use could allow practitioners to help their patients derive value from available technologies. Demographic differences in eHealth use indicate that groups that have traditionally been able to effectively navigate the healthcare system (ie, wealthier or more highly educated individuals) are best positioned to use eHealth resources available to them [14-18]. Consequently, these groups may be most likely to use eHealth in response to a problem in their care, thereby limiting the protective effect of broad access to eHealth tools.

This study aimed to determine the relationships between patients’ experiences of problems with the healthcare system and the use of varied eHealth tools. We first classified the eHealth activities assessed in the 2017 Health Information National Trends Survey (HINTS) into related groups. We then used these factors to determine how two types of negative care experiences (ie, low patient centeredness and care coordination problems) motivated the use of different kinds of eHealth activities and whether education level affected the associations between eHealth use and negative experiences.

## Methods

### Data

We used data from the first wave of the 2017 HINTS, which is a cross-sectional, nationally representative survey of American adults. HINTS is designed to analyze how people use health information, with a focus on information technology and healthy behaviors. We selected the HINTS data because they contain unique information on people’s interactions with the healthcare system and their use of eHealth tools.

### Population

Our study included individuals aged ≥18 years in the civilian non-institutionalized population of the United States. Respondents were excluded from analyses if they were missing >25% of data in the measures of eHealth activity (n=143), patient centeredness (n=500), or care coordination (n=30). Thus, our final sample included data from 2612 respondents.

### Dependent Variable: eHealth Activity

There are many available eHealth activities, and studies frequently select only one or a few activities for analysis. The HINTS survey includes 25 items related to eHealth activity across 4 instruments. Instead of limiting the activities in our analyses, we categorized these tools into conceptually similar groups. We used exploratory factor analysis to identify the number of underlying constructs onto which eHealth items loaded. These analyses, described in detail in the Analysis section below, resulted in the construction of two dependent variables: eHealth activities used to communicate with healthcare providers (11 provider-facing activities) and eHealth activities performed independent of the provider (10 independent activities).

### Independent Variable: Negative Healthcare Experiences

Two sets of survey items in HINTS are related to negative healthcare experiences. *Patient centeredness* of care was measured using a 7-item scale based on the core functions of patient-centered communication identified by Epstein and Street [19] and widely used in past research [19,20]. Respondents were asked 7 questions about how often (Always, Often, Sometimes, Never) providers (1) “Give you the chance to ask all the health-related questions you had,” (2) “Give the attention you needed to your feelings and emotions,” (3) “Involve you in decisions about your healthcare as much as you wanted,” (4) “Make sure you understood the things you needed to do to take care of your health,” (5) “Explain things in a way you could understand,” (6) “Spend enough time with you,” and (7) “Help you deal with feelings of uncertainty about your health or health care.” To create a summary of responses, we calculated the mean of each respondents’ answers to all 7 questions. As the responses were highly skewed, we operationalized this variable as tertiles rather than as a continuous measure. The tertiles represented relatively low, medium, and high patient centeredness. If the patient reported low or medium patient centeredness, they were considered to have *negative experiences of patient centeredness*. This conceptual categorization of perceived patient centeredness into very-positive versus less-positive perceptions is consistent with the methods of previous studies that used this measure and other measures of patient-centered communication [7,20,21].

The second set of survey items focused on *problems in care coordination*. Four survey items on this concept were included in HINTS. Respondents were asked whether at some point in the last 12 months, they (1) “Had to bring an X-ray, MRI, or other type of test result with you to the appointment,” (2) “Had to wait for test results longer than you thought reasonable,” (3) “Had to redo a test or procedure because the earlier test results were not available,” and (4) “Had to provide your medical history again because your chart could not be found.” We excluded the survey item about bringing a test result to an appointment because we believed it lacked face validity; unlike the other 3 items, this item was not considered problematic. Therefore, we were concerned that any patient who underwent imaging might answer this question positively. In support of this reasoning, during initial data cleaning, we empirically observed that this “problem” was reported far more often (572/2612, 22% unweighted, 19% weighted) vs an average of 225/2612 (8.6% unweighted, 9.4% weighted) respondents for the other 3 problems) and that correlations between this “problem” and the other 3 problems were low (0.17 on average). Because each item was relatively rare in the initial data analysis, we operationalized this variable dichotomously. If the patient experienced at least 1 of the 3 problems, they were considered to have *negative experiences of care coordination.*

### Stratifying Variables

In the HINTS data, education is measured on a 5-point scale: (1) Less than high school, (2) High school graduation, (3) Some college, (4) Bachelor’s degree, and (5) Postbaccalaureate degree. We stratified the sample into higher and lower education levels to determine whether these groups engaged in eHealth differently when they had negative healthcare experiences. The median level of education was some college experience, and more respondents reported a college or higher education level than a high school or lower education level (30.2% vs 36.1%). Therefore, we categorized participants with education level lower than a bachelor’s degree as having a lower education level and those with a bachelor’s or postbaccalaureate degree as having a higher education level. A subsequent sensitivity analysis grouped participants with at least some college experience along with participants with a higher education level.

### Control Variables

We included several variables in our multivariate analysis to account for factors that may introduce bias in the relationships between negative healthcare experiences and the use of eHealth. We included 4 demographic variables (race, gender, age, and income), two variables related to use of the internet (whether they ever use the internet and whether they accessed the internet from home), and patients’ self-reported general health, each of which may be associated with both the extent to which individuals experience problems with their healthcare and their use of eHealth resources.

### Analysis

#### Factor Analysis

To test the first research question, that is, how eHealth activities can be categorized on the basis of their usage, we used exploratory factor analysis with oblique promax rotation. We chose this rotation because we did not want to constrain the data based on an assumption of orthogonality. We retained factors with an eigenvalue >1, which is the typical cutoff to retain factors for analysis. Items were excluded if, following rotation, they did not load onto any factor at levels >0.40 or if they loaded onto multiple factors at levels >0.40.

#### Regression Analysis

We created two multivariate generalized linear model regressions for our second research question about the relationship between negative healthcare experiences and eHealth activities. In one model, we estimated how negative healthcare experiences (medium or low perceptions of patient centeredness and experience of at least one coordination problem) were associated with the use of provider-facing eHealth activities. In the second model, we analyzed the associations between these two negative healthcare experiences and independent eHealth activities. We included covariates related to demographics, internet use, and general health in each model. We used negative binomial regressions because the outcomes were counts of eHealth activities and were overdispersed. We used survey weights to ensure that our estimates were representative of the US population.

We divided our sample into two groups according to higher and lower education levels to address our third research question about whether the relationships between negative healthcare experiences and eHealth activity differed across educational levels. Subsequently, we recreated the two negative binomial regression models described above in this section to estimate the relationships between negative healthcare experiences and provider-facing and independent eHealth activities within each education group. Finally, we plotted the predicted level of each eHealth activity based on negative healthcare experiences to facilitate comparison of the magnitude of effects. All statistical analyses were conducted in Stata 16 MP (Stata Corporation, College Station, TX).

## Results

### Summary Statistics

Our final study sample included 2612 individuals (Table 1), of which 63.5% (survey-weighted) were non-Hispanic white, 9.2% were Hispanic, and 13.1% were non-Hispanic African-American. In addition, 62.6% of respondents (weighted) did not receive a bachelor’s or higher degree. The mean age of the study population was 49 years, and the modal health rating was “Very good.”

### Factor Analysis

Only two factors had eigenvalues >1. Following oblique promax rotation, 21 of the 25 eHealth activity items loaded clearly onto one of the two factors, which together accounted for 93% of the variance in reported eHealth use. The first factor included 11 eHealth activities used to communicate with healthcare providers (provider-facing activities). The second factor included 10 eHealth activities independent of the provider (independent activities; Table 2). The remaining 4 items were excluded from analyses because they failed to load onto either factor using the factor-loading cutoff of .40. Use of provider-facing activities and independent activities were positively correlated with each other (*r* [2,612]=.48). A mean comparison using *t*-test showed that respondents had used fewer provider-facing activities (mean 1.94, SD 2.70) than independent activities (mean 3.75, SD 2.71; *t* [2611]=33.42, *P*<.001, 95% CI 1.70-1.92).

### Regression Analysis

#### Full Sample

Overall, neither problems in care coordination nor perceived patient centeredness predicted the number of provider-facing activities used (Table 3). In contrast, participants who experienced problems with care coordination used an average of 0.50 (14.9%) more independent eHealth activities than those who did not experience such problems (beta=1.15, *P*=.01). Compared to participants who perceived high levels of patient centeredness, those who perceived moderate levels of patient centeredness used an average of 0.44 (14.0%) more independent activities (beta=1.14, *P*=.02) and those who perceived low levels used an average of 0.50 (15.9%) more independent activities (beta=1.16, *P*=.01).

#### Education-Stratified Groups

**Figure 1:**
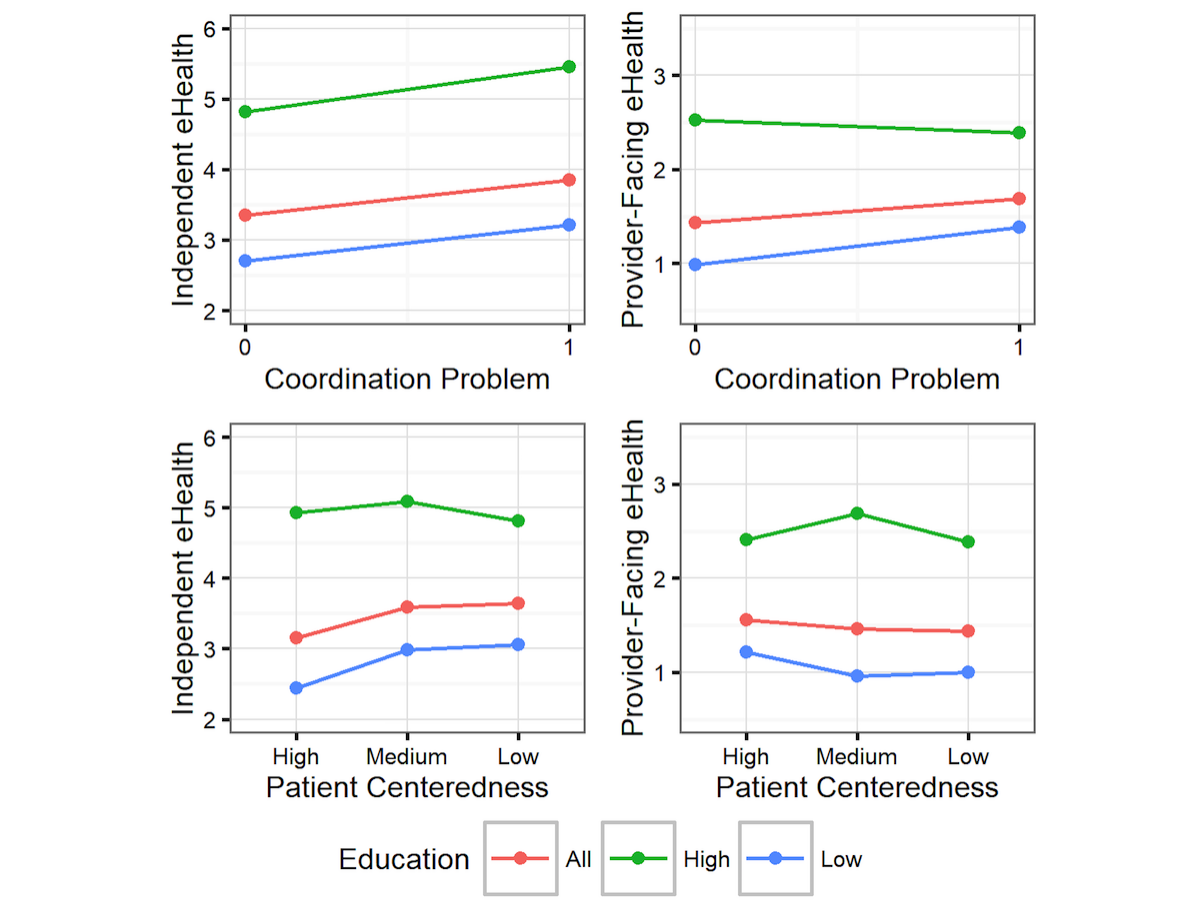
Associations between healthcare experiences and eHealth use, stratified by education level.

Provider-facing eHealth activity was not predicted by problems in care coordination or perceived patient centeredness in the model restricted to more highly educated adults (Figure 1). Among individuals with education below college level, those who experienced problems with care coordination used an average of 0.40 (40.4%) more provider-facing eHealth activities than those who did not experience such problems (beta=1.40, *P*=.07). However, this finding should be interpreted with caution, as it was not significant in our sensitivity analysis (ie, when participants with some college education were categorized as having higher education levels, Multimedia Appendix 1). The perceived lack of patient centeredness remained nonsignificantly associated with provider-facing eHealth use among adults with lower levels of education.

In the stratified model restricted to more highly educated individuals, problems with care coordination were associated with the use of 0.63 (13.1%) more independent eHealth activities (beta=1.13, *P*=.009), whereas perceived patient centeredness was not associated with the use of these activities. Among individuals with education below college level, those who experienced problems with care coordination used an average of 0.51 (18.8%) more provider-facing eHealth activities than those who did not experience such problems, showing a marginally significant increase (beta=1.19, *P*=.07). This finding should also be interpreted with caution, as it was nonsignificant in our sensitivity analysis (Multimedia Appendix 1). Compared to participants who perceived high levels of patient centeredness, those who perceived moderate levels of patient centeredness used an average of 0.55 (22.4%) more independent activities (beta=1.22, *P*=.02) and those who perceived low levels used an average of 0.62 (25.4%) more independent activities (beta=1.25, *P*=.02).

**Table 1:** Survey-weighted summary characteristics of the 2017 Health Information National Trends Survey respondents in current analyses.

Variable		n (%)	95% CI
**Education**			
	Less than high school	148 (7.5)	5.5-9.5
	High school graduate	467 (22.7)	20.3-25.1
	Some college	752 (32.4)	30.0-34.7
	Bachelor’s degree	697 (22.2)	20.7-23.6
	Postbaccalaureate degree	491 (13.9)	12.6-15.3
**Race/ethnicity**			
	Non-Hispanic white	1568 (63.5)	61.3-65.8
	Hispanic	333 (9.2)	8.1-10.3
	Non-Hispanic African-American	296 (13.1)	11.6-14.5
	Non-Hispanic Asian	95 (4.4)	3.7-5.1
	Other	98 (2.8)	2.4-3.2
**Gender**			
	Male	996 (45.6)	43.8-47.4
	Female	1580 (53.5)	51.7-55.3
**Age**			
	18-34 years	280 (21.3)	18.0-24.5
	35-49 years	514 (26.7)	23.3-30.2
	50-64 years	877 (30.0)	28.0-32.1
	65-74 years	563 (11.5)	10.8-12.1
	≥75 years	292 (7.8)	7.2-8.5
**Income (USD)**			
	$0-$34,999	805 (28.6)	25.7-31.4
	$35,000-$100,000	1125 (44.5)	41.0-48.1
	≥$100,000	660 (25.8)	23.1-28.4
**General health**			
	Poor	64 (2.4)	1.4-3.4
	Fair	410 (14.5)	12.3-16.7
	Good	903 (34.1)	30.5-37.6
	Very good	944 (37.3)	33.9-40.8
	Excellent	266 (10.8)	8.3-13.4
**Use Internet**			
	No	499 (16.4)	14.5-18.2
	Yes	2113 (83.6)	81.8-85.5
**Use Internet at home**			
	Not applicable	100 (4.4)	3.0-5.9
	Never	482 (16.4)	14.4-18.3
	Sometimes	627 (25.2)	22.6-27.7
	Daily	1260 (49.8)	46.7-52.9

**Table 2:** Factor analysis results.

Item		Factor loadings	
		Provider-Facing eHealth	Independent eHealth
**In the past 12 months, have you used a computer, smartphone, or any other electronic means to do any of the following?**			
	Look for health or medical information for yourself	–0.04	.62
	Look for health or medical information for someone else	–0.06	.58
	Buy medicine or vitamins online	.08	.28
	Look for a healthcare provider	–0.04	.55
	Use e-mail or the Internet to communicate with a doctor or a doctor’s office	.44	.31
	Make appointments with a health care provider	.29	.31
	Track healthcare charges and costs	.20	.43
	Fill out forms or paperwork related to your health care	.16	.44
	Look up test results	.56	.23
**In the past 12 months, have you used your online medical record to**			
	Make appointments with a healthcare provider	.74	–0.02
	Fill out forms or paperwork related to your healthcare	.62	.07
	Request refill of medications	.72	–0.07
	Request correction of inaccurate information	.39	–0.03
	Add health information to share with your healthcare provider, such as health concerns, symptoms, and side-effects	.60	–0.06
	Download your health information to your computer or mobile device such as a cell phone or tablet	.48	–0.01
	Help you make a decision about how to treat an illness or condition	.57	–0.03
	Securely message a health care provider and staff (eg, e-mail)	.80	–0.04
	Monitor your health	.66	–0.05
	Look up test results	.76	.03
**In the last 12 months, have you used the Internet for any of the following reasons?**			
	To share health information on social networking sites such as Facebook or Twitter	–0.07	.41
	To participate in an online forum or support group for people with a similar health or medical issue	–0.05	.35
	To watch a health-related video on YouTube	–0.06	.52
**Has your tablet or smartphone**			
	Helped you track progress on a health-related goal such as quitting smoking, losing weight, or increasing physical activity	.05	.46
	Helped you make a decision about how to treat an illness or condition	–0.07	.58
	Helped you in discussions with your healthcare provider	.09	.51

**Table 3:** Weighted negative binomial regression predicting provider-facing and independent eHealth activities.

Variable		Provider-facing eHealth		Independent eHealth	
		Incidence rate ratios (SE)	*P* value	Incidence rate ratios (SE)	*P* value
Care coordination problem		1.18 (0.14)	.16	1.15 (0.06)	.01
**Patient centeredness (reference: High)**					
	Medium	0.94 (0.10)	.58	1.14 (0.06)	.01
	Low	0.92 (0.13)	.57	1.16 (0.07)	.01
**Race/ethnicity (reference: Non-Hispanic white)**					
	Hispanic	0.98 (0.16)	.90	1.03 (0.08)	.67
	Non-Hispanic African-American	0.84 (0.14)	.29	1.01 (0.05)	.91
	Non-Hispanic Asian	1.14 (0.27)	.58	1.23 (0.09)	.01
	Other	1.01 (0.23)	.95	1.15 (0.13)	.22
	Missing	1.18 (0.31)	.53	1.24 (0.27)	.33
**Gender (reference: Male)**					
	Female	1.22 (0.15)	.12	1.16 (0.06)	.004
	Missing	0.92 (0.38)	.85	1.31 (0.29)	.22
**Age (reference: 18-34 years)**					
	35-49 years	1.32 (0.22)	.09	0.95 (0.07)	.47
	50-64 years	1.17 (0.19)	.35	0.77 (0.06)	.001
	65-74 years	1.06 (0.18)	.74	0.65 (0.06)	<.001
	≥75 years	0.98 (0.22)	.91	0.43 (0.05)	<.001
	Missing	1.01 (0.43)	.98	0.46 (0.14)	.01
**Education (reference: Less than high school graduate)**					
	High school graduate	1.31 (0.39)	.36	1.12 (0.23)	.57
	Some college	1.72 (0.55)	.09	1.31 (0.27)	.20
	Bachelor’s degree	2.28 (0.73)	.01	1.44 (0.29)	.08
	Postbaccalaureate Degree	2.14 (0.71)	.03	1.38 (0.28)	.12
	Missing	0.85 (0.41)	.74	1.01 (0.41)	.98
**Income (USD; reference: $0-$34,9999)**					
	$35000-$99,9999	1.39 (0.31)	.14	1.14 (0.06)	.02
	≥$100,000	1.59 (0.37)	.05	1.21 (0.08)	.009
	Missing	0.93 (1.01)	.94	0.96 (0.54)	.94
**General health (reference: Poor)**					
	Fair	0.98 (0.57)	.97	1.29 (0.31)	.28
	Good	0.78 (0.46)	.68	1.30 (0.33)	.31
	Very good	0.92 (0.54)	.89	1.34 (0.33)	.23
	Excellent	0.81 (0.47)	.72	1.37 (0.34)	.21
	Missing	0.36 (0.19)	.06	1.35 (0.44)	.37
Use Internet		2.70 (0.87)	<.001	2.41 (0.39)	<.001
**Use Internet at home (reference: Not Applicable)**					
	Never	2.15 (1.01)	.11	1.27 (0.22)	.17
	Sometimes	2.29 (0.84)	.03	1.48 (0.17)	.001
	Daily	2.59 (0.92)	.01	1.55 (0.17)	<.001
	Missing	1.44 (0.72)	.47	1.38 (0.24)	.07
	Constant	0.11 (0.10)	.11	0.71 (0.22)	.15

## Discussion

We examined the relationships between negative experiences with the healthcare system and the use of eHealth, and whether these relationships differed across individuals’ education levels in a nationally representative sample of adults. We found that eHealth activities were clearly divided into two categories: provider-facing activities (facilitating access to providers and communication with providers) and independent activities (patient-driven information seeking and communication with non-providers). Negative care experiences were not associated with provider-facing eHealth activity in the overall population or among more highly educated respondents; however, respondents with a lower education level were more likely to use these activities if they experienced problems with care coordination. These results were different for independent eHealth activity: Overall, individuals were more likely to engage in these activities if they experienced problems with either care coordination or patient-centered communication. Although care coordination problems predicted independent eHealth activity similarly across education levels, the relationship between low perceived patient centeredness and independent activity seemed limited to individuals with lower levels of education. The cross-sectional nature of these data precludes us from determining whether eHealth use results from these negative care experiences; however, our findings suggest that people may use eHealth to address deficiencies in healthcare, and this potential protective effect is more pronounced in groups that have traditionally struggled to navigate the healthcare system (individuals with lower levels of education).

The two underlying categories we identified using factor analysis resonate with existing literature on eHealth, which tend to focus on provider-facing eHealth tools or independent health information seeking, but rarely on both [6]. Existing individual analyses focused on one or a few eHealth activities, making the comparison of results across studies difficult (eg, [14] vs [16]). The structure we identified provides a framework for determining how the use of one kind of activity might affect the use of other activities. In addition, our approach facilitates measurement of multiple kinds of eHealth activities concurrently and limits potentially arbitrary selection of eHealth activities for analysis. Finally, scale construction facilitates investigation of the intensity of eHealth use. As internet access and advanced electronic health records become increasingly widespread, binary indicators of eHealth use may become less meaningful and measures of intensity may become more important [22].

Mean levels of eHealth activity indicate that these resources remain underused, with people using, on average, less than half of the eHealth resources available to them. Provider-facing activities are especially underused, indicating the need for researchers and healthcare professionals to identify and remedy barriers to their adoption and use. Although eHealth activities are overall underused, the current results suggest greater use among individuals with negative healthcare experiences than in those without such experiences. Similar to previous studies, we found that perceived low patient centeredness predicted increased independent activity [7,10]. Extending this prior work, we observed that care-coordination problems were associated with greater independent eHealth use. In contrast to these effects for independent use, neither low patient centeredness nor care coordination problems contributed to provider-facing use in this combined, nationally representative sample. This may indicate that, when individuals experience problems with care, eHealth activities that act as *alternatives* to the traditional healthcare system may seem more useful than tools that improve interactions with the system.

Contrary to our expectations, people with lower levels of education may be more likely to seek alternatives or supplements when care problems occur as compared to individuals with higher levels of education. This suggests a potential protective effect for a disadvantaged group (individuals with lower levels of education), as they seek alternatives or supplements when care problems occur. In particular, our findings suggest that individuals with lower education levels may react more to problems with patient-centered communication than individuals with higher education levels. Despite this relationship, individuals with lower education levels used eHealth resources at lower rates than those with higher education levels. This finding is consistent with a persistent digital divide in eHealth use associated with other health disparities [14,17]. These findings indicate that eHealth could help address differences in the quality of care received by different socioeconomic groups, but new strategies are needed to increase its adoption and use in vulnerable populations if these resources are to meet their potential of reducing health disparities [18]. Future work should focus on ensuring equitable access to eHealth resources as well as the creation and dissemination of culturally appropriate eHealth tools.

Our study has several limitations. First, the construction of the HINTS survey may have contributed to sorting of individual items in the factor analysis, as responses to nearby items on instruments are likely correlated by construction. Although some survey instruments loaded fully onto one factor, others contributed items to both or neither category, and the survey construction alone did not fully explain the pattern of our results. Future work should aim to replicate these results in other surveys. Second, our analyses were cross-sectional in nature. We observed associations between negative healthcare experiences and eHealth use and hypothesized that patients use eHealth in response to these care experiences, but our data cannot support this causal inference. As such, our results are subject to potential bias or reverse causality. One possible source of bias is that people with more complex health problems may be more likely to use provider-facing tools and experience coordination problems. To reduce the potential for bias, we included a set of patient demographics, internet access, and health status variables to control for the observed differences in respondents. Finally, although we discuss eHealth as a promising resource, we were unable to test whether its use improves health in individuals with negative care experiences. Measuring the impact of eHealth on outcomes and developing strategies to maximize the potential benefits of eHealth remain important areas of study but are beyond the scope of this research.

A strength of the current work is that the data were sourced from a nationally representative sample of Americans. However, we should ascertain whether these results can be replicated in other cultural contexts. People in “Western, Educated, Industrialized, Rich, and Democratic (WEIRD)” societies like the United States are frequent outliers in behavioral research [23], with a strong focus on independence and autonomy. However, Americans with lower levels of education, similar to the worldwide population, tend to value interpersonal connection more strongly [23,24]. This may partially explain the increased responsiveness to deficits in the patient-provider relationship among individuals with lower education levels. It is possible that the overall patterns of eHealth motivation more closely resemble those of more highly educated Americans in cultural contexts that value independence and those of less highly educated Americans in cultural contexts that value interpersonal connection. Compared to populations of other nations, Americans face shorter wait times to visit their providers. The use of eHealth tools in response to negative care experiences may be more prevalent in nations where followup visits to address these experiences are limited [25]. Therefore, it is possible that the trends observed in this study may be more pronounced in other settings.

Our findings indicate that individuals use eHealth activities, especially those that are independent of healthcare providers, when they experience problems with their healthcare. In particular, individuals with lower levels of education seem to use eHealth in response to negative healthcare experiences. Nonetheless, eHealth use remains low overall, and eHealth is an underused means of improving health outcomes. To maximize the potential for eHealth to meet the needs of all patients, especially those who are traditionally underserved by the healthcare system, additional work should ensure that eHealth resources are accessible to and usable by all members of the population.

## Appendix

Multimedia Appendix 1 Sensitivity testing for stratified regression analyses, with "Some college" categorized as higher education.

## Abbreviations

HINTS: Health Information National Trends Survey
WEIRD: Western, Educated, Industrialized, Rich, and Democratic
